# Localization of the Autism Spectrum Disorder Knowledge Scale Professional Version (ASKSP-R) in western cities of China

**DOI:** 10.3389/fpsyt.2025.1550823

**Published:** 2025-09-10

**Authors:** Yueying Zhang, Yang Hong, Zhujun Zhao, Fang Hou, Feng Hong, Fudong Li

**Affiliations:** ^1^ School of Public Health, The Key Laboratory of Environmental Pollution Monitoring and Disease Control, Ministry of Education, Guizhou Medical University, Guiyang, China; ^2^ Guizhou Nursing Vocational College, Guiyang, China

**Keywords:** autism spectrum disorder, knowledge, autism spectrum knowledge scale for professional version-revised scale, chinese, professional population

## Abstract

**Purpose:**

This study aimed to translate, revise, and validate the Autism Spectrum Knowledge Scale for Professional Version-Revised (ASKSP-R), used to assess the knowledge of professionals involved in autism spectrum disorder (ASD) care and services, such as such as clinicians, educators, and therapist, in the Chinese context.

**Methods:**

The Chinese version of ASKSP-R demonstrated high internal consistency (Cronbach’s α = 0.885) and strong structural validity (KMO = 0.888, RMSEA = 0.059). CFA supported a multidimensional structure, with acceptable fit indices (e.g., IFI = 0.88). IRT analysis showed suitable difficulty (-3 to +3) and discrimination (>0.5) parameters for all items.

**Conclusion:**

The revised ASKSP-R scale exhibited robust psychometric properties, and it can help assess the relevant knowledge of clinicians, educators, therapists, and other ASD-related professionals, enabling appropriate interventions based on assessment results, promoting targeted training and education, and increasing the rate of early diagnosis and intervention for ASD.

## 1 Introduction

Autism Spectrum Disorder (ASD) was first proposed by Kanner (1). The Diagnostic and Statistical Manual of Mental Disorders, Fifth Edition (DSM-5™), specifies persistent deficits in social communication and interaction, as well as restricted and repetitive behaviors as the main characteristics of ASD (2). Recently, the global prevalence of ASD has been increasing significantly, and approximately 1% of children have been diagnosed with ASD (3). Recent epidemiological updates indicate over 12.7 million ASD diagnoses in China, including 2.4 million children under 12 years, with incidence rising at >200,000/year (3, 4). Despite the increasing prevalence of ASD, the issue of delayed diagnosis persists. While the average age of diagnosis is 4–5 years, the majority of ASD children do not undergo developmental assessment before the age of 3 years (5, 6, Keen & Ward’s 7, 8). Delayed diagnosis prevents young children to be benefited from their optimal neuroplasticity period. Contemporary studies confirm persistent global diagnosis delays averaging 28.5 months post-symptom onset (9), exacerbating long-term functional outcomes. In addition to missing out on the ideal time to intervene, it also increases the burden on both individuals and society (10). Hence, early diagnosis and interventions have significantly improved the prognosis of ASD children and reduced long-term social expenditure (9, 11, 12). Moreover, a lack of expertise in relevant professionals can cause misdiagnosis or delayed diagnosis and slow down the patient’s recovery process [13 Clarke and Fung (14), 15)]. However, ASD knowledge in the general Chinese population is low. Although the level of ASD knowledge among medical professionals is particularly important, there is currently no assessment tool for understanding the ASD knowledge level among Chinese professionals (4). Thus, a comprehensive assessment tool is required to understand and improve ASD knowledge among concerned professionals. In the Chinese context, ASD diagnosis and intervention involve a multi-disciplinary approach. Screening mainly begins with pediatricians during routine health checkups, followed by referrals to psychologists or neurologists for further evaluation. Educational and rehabilitation professionals, such as special education teachers, are essential in implementing interventions tailored to the child’s needs. Although formal pre-qualification training in ASD is not universally required for all professionals, psychologists and medical practitioners typically receive some training as part of their professional education. However, the depth of this training varies widely, and additional professional development is often needed to ensure adequate knowledge and skills for ASD-related tasks.

In recent years, domestic studies on ASD knowledge have mainly concentrated on the general population. Few studies have targeted ASD professionals, and most of the existing questionnaires are self-developed questionnaires (16) and lack a scientific basis. A few translated foreign scales are available for the general population (17). Very few ASD knowledge scales have been developed for relevant professionals. The general Chinese population has limited awareness of ASD (18), with the majority unaware that effective interventions and support strategies are available for individuals with ASD (17). Furthermore, many people are unaware of where skill-building interventions or specialized training programs for ASD are most effectively implemented (19). A study by Lu et al. (19) indicated that approximately 80% of parents choose “wait and see” if their child develops abnormal behaviors as they grow older. Alyami et al. (20) suggested that the least ASD knowledge in the general population was in the areas of ASD etiology. However, Lu et al. (19) revealed that the general population is aware that children should be taken to the hospital after they develop abnormalities. A majority of them also consider that ASD requires long-term skill-building interventions or specialized training programs, and its symptoms can be improved by long-term training. Presently, the questionnaire used to measure the ASD knowledge of the general Chinese population is the Autism Stigma and Knowledge Questionnaire (ASK-Q), which modified by Lodi et al. This questionnaire has been widely used domestically and internationally (18, 21). The majority of the general population is unaware of the primary symptoms of ASDs, ASD co-occurring intellectual disability, and prognosis (18). Gu et al.’s questionnaire, exclusively for kindergarten healthcare providers, did not include a scale for ASD professionals (22). Although China has various ASD knowledge questionnaires, most of these questionnaires are aimed at the general population or caregivers, and there is a lack of ASD scales specifically for professionals (4, 17, 18, 23, 24).

Research on ASD in foreign countries remains in its early stages, with a particular concentration on the knowledge of ASD professionals. Several international questionnaires have been developed to assess ASD knowledge, including the Knowledge about Childhood Autism among Health Workers (KCAHW) by McMahon et al. (25), the Autism Knowledge Scale by Unigwe et al. (16), the Autism Knowledge Scale by Crane et al. (26), and the Autism Spectrum Knowledge Scale Professional Version-Revised (ASKSP-R) by McClain et al. (27). The Autism Knowledge Scale by Unigwe et al. (16) is a relatively new instrument based on an earlier scale by Zwaigenbaum et al. (12). However, it provides only “correct” and “incorrect” response options, omitting a “don’t know” choice, which limits its ability to fully capture a doctor’s actual ASD knowledge. While the scale by Crane et al. (26) is more comprehensive than that of Unigwe et al. (16), it lacks a rehabilitation component, is more cumbersome to use, and demands greater effort. The KCAHW questionnaire, developed by Bakare et al. (28), is widely used and frequently referenced. However, it lacks coverage of diagnostic aspects and does not provide sufficient detail for specialized medical professionals. In contrast, the ASKSP-R scale is praised for its scientific rigor and comprehensiveness, although no Chinese version is currently available.

The ASKSP-R scale for ASD professionals, developed by McClain et al. (27), provides comprehensive knowledge coverage, demonstrates strong reliability and validity, and aligns with the DSM-5 by addressing multiple disease content areas. To reduce guesswork, the scale includes a “do not know” response option alongside the traditional “correct” and “incorrect” choices (25). However, a Chinese version of the scale has not yet been developed (27). Consequently, the scale has been translated for this purpose. While there is no cure for autism, evidence-based interventions, such as behavioral therapies, educational support, and targeted skill-building strategies, can significantly improve outcomes and quality of life for individuals with ASD. The system compared the differences between ASKSP-R and the existing scales. For example: The KCAHW questionnaire: It only targets medical staff and does not cover education/rehabilitation practitioners; The Crane scale: It lacks the dimension of rehabilitation intervention and does not incorporate the DSM-5 diagnostic criteria; The advantages of ASKSP-R: It covers four dimensions of diagnosis, symptoms, etiology, and treatment, includes an “unknown” option to reduce guesswork bias, and meets the needs of multi-disciplinary collaboration in China (**Table 1**).

**Table 1:** Comparative analysis of ASD knowledge assessment tools.

Scale	Target group	Cultural validity	DSM-5 alignment	“Don’t know” option	Psychometric strength
ASKSP-R	Multidisciplinary	TRAPD-validated	Full	Included	α=0.885, RMSEA=0.059
Bakare et al (26)	Health workers	Nigeria-specific	Partial	Absent	α=0.72
Crane et al (26)	Psychiatrists	UK-focused	Limited	Absent	Not reported
Unigwe et al (16)	General Practitioners	No adaptation	No	Absent	α=0.68

This study aimed to translate and adapt the ASKSP-R scale to assess ASD professional knowledge in China through localized revisions. It also aimed to evaluate the current knowledge levels of Chinese ASD professionals, identify factors influencing their understanding of ASD, and develop targeted training programs to enhance their expertise. These efforts are expected to improve early diagnosis and intervention for ASD, thereby enhancing affected patients’ quality of life.

## 2 Methods

### 2.1 Scale revision

After translation, the scale was revised using the TRAPD (Translation, Review, Adjudication, Pretesting, and Documentation) model, with consent from the original authors (29). Firstly, two professional translation teams independently translated and reviewed the scale. A third qualified translator then reviewed the translation, conducted an accuracy check, and completed the Reviewer’s Spreadsheet. The TRAPD framework was further validated using NLP-powered semantic analysis of back-translations (30), achieving 92.4% conceptual equivalence. Additionally, the scale was modified through expert consultations and a literature review. Two senior ASD experts, with extensive experience in both research and clinical practice, were consulted to refine the questionnaire content. Their input was crucial for adapting the scale to the Chinese context and ensuring its localization. A pre-survey was conducted to assess the scale’s feasibility and comprehensibility in real-world use. Based on the pre-survey feedback, several questions were revised to enhance the scale’s clarity and applicability. The revised version was again reviewed and confirmed by the ASD experts, resulting in the final Chinese version of the ASKSP-R scale. Based on the experts’ comments and relevant policy literature, the modifications were made to the ASKSP-R scale, which were derailed in **Supplementary File**. The ASKSP-R uniquely addresses China’s multidisciplinary ASD management model encompassing pediatricians, neurologists, special educators, and rehabilitation therapists. Unlike existing tools focusing on single professions, its four-domain structure (Etiology/Epidemiology, Symptoms/Behaviors, Assessment/Diagnosis, Treatment) reflects DSM-5 diagnostic criteria while incorporating culturally adapted interventions (29).

### 2.2 Study subjects

Study participants included physicians (e.g., pediatricians, neurologists) responsible for ASD screening and diagnosis, and special education teachers involved in intervention and rehabilitation. The study aimed to include a mix of professionals from both specialist and general settings, such as hospitals and special education schools, to capture a wide range of expertise. However, professionals in specialist schools may work predominantly with children with more severe needs, which could influence their perspectives and knowledge. Future research could consider stratified sampling to better represent different professional subgroups and settings. Professionals involved in ASD diagnosis and care in China include psychologists, who provide assessments and initial diagnoses, medical practitioners (e.g., pediatricians, neurologists, child health doctors) responsible for confirming diagnoses and developing intervention plans tailored to the individual’s needs and co-occurring conditions, and special education teachers, who contribute to intervention and rehabilitation programs.

The scale was based on random and snowball sampling methods. A total of 2,550 questionnaires were distributed, and 2548 valid questionnaires were returned with a recovery rate of 99.92%. Our study population comprised teachers ≥ 18 years old, working in special education schools, and physicians directly involved in ASD screening, diagnosis, and intervention. Those with incomplete questionnaires or who did not provide informed consent were excluded to ensure the data’s accuracy and reliability. **Table 2** lists the participants’ demographic information, including age, gender, education, title, ethnicity, if they have interacted with ASD children, if they know about ASD, and how they learned about it. **Table 2** depicts patients’ demographic information.

**Table 2:** Patients’ demographic data.

Considerations	Groups	Number of participants N=2548(%)
(a person’s) age	18-25 years	202(7.93)
	26-35 years	1462(57.38)
	36-45 years	664(26.06)
	46-55 years	192(7.54)
	55-60 years	25(0.98)
	60 years and over	3(0.12)
distinguishing between the sexes	male	679(26.65)
	female	1869(73.35)
academic qualifications	Technical secondary school and below	6(0.24)
	College/Undergraduate	2434(95.53)
	Postgraduate and above	108(4.24)
title	none	302(11.85)
	Primary title	1164(45.68)
	Middle title	810(31.79)
	Vice-senior title	206(8.08)
	Senior title	66(2.59)
nation	Han ethnic group	1491(58.52)
	Miao ethnic group	313(12.28)
	Tujia ethnic group	136(5.33)
	Dong ethnic group	147(5.76)
	Buyi ethnic group	174(6.82)
	the rest	287(11.24)
Exposure to children with ASD	Yes	1190(46.70)
	No	1358(53.30)
Whether you have learnt about ASD	Yes	1015(39.84)
	No	1533(60.16)
In what way do you learn about ASD	Self-study	625(24.53)
	I learnt it in school.	464(18.20)
	Unified on-site training from company	379(14.86)
	Unified online training from company	322(12.62)
	the rest	152(5.96)

### 2.3 Data collection and procedures

We collected data from April 2024 to May 2024. The specific steps were: Pre-survey period: A pre-survey was conducted from 13th to 18th February 2024 with 32 participants, including a mix of medical professionals and special education teachers, to evaluate the feasibility, clarity, and cultural relevance of the translated scale. Participant feedback from the pre-survey highlighted areas where question phrasing needed to be simplified or localized. These changes were essential to ensure that the scale was understandable and relevant to Chinese professionals. The finalized scale was further tested for consistency and clarity before full-scale data collection. Feedback from this phase identified ambiguities in certain questions and informed adjustments to improve clarity and applicability. For instance, terminology inconsistencies and region-specific content were refined to better align with Chinese professionals’ practices. These modifications were reviewed and approved by two senior ASD experts.

Preparation period: We contacted hospital directors to ensure precise questionnaire distribution and data collection. Regarding the questionnaire distribution, the ASKSP-R scale (Chinese version) post-final modification was distributed to ASD-related departments like pediatrics, rehabilitation, neurology, psychiatry, and schools for special children after obtaining the consent of the head of the hospital. The head of each department and school was responsible for administering the questionnaire and explaining it to the participants.

Before starting the survey, the questionnaire mentioned the study’s purposes, estimated filling time, and the content of the informed consent form. Participants filled the questionnaire voluntarily to ensure their right to information and willingness to participate.

Data were collected using random sampling and snowball sampling. Firstly, several hospitals were randomly selected for our survey. Subsequently, the questionnaire’s QR code was sent to the interviewed doctors and special education teachers through the ‘Questionnaire Star’ platform. This was further shared with other professionals for a snowball effect and to expand the sample coverage. Considering the extensive geographical distribution of the participating hospitals and the target population’s workload, we adopted an electronic questionnaire format and unified data collection through the ‘Questionnaire Star’ platform (https://www.wjx.cn/login.aspx). The ‘Questionnaire Star’ platform is a free online questionnaire survey and evaluation platform that includes an efficient and convenient online questionnaire design and data collection method and is widely used in several Chinese studies. We utilized the AI-enhanced ‘Questionnaire Star 4.0’ (4) featuring adaptive test branching based on real-time ability estimation (31). In this platform, researchers can design questionnaires online and independently, with the integration of questionnaire quantity, time, and location. Being more efficient and convenient, it is widely used in China. Before beginning the survey, the heads of the participating hospitals and schools were responsible for facilitating the distribution of the questionnaires and providing general information to participants. However, the consent process was strictly individual. Participants were provided with detailed information about the study’s purpose, the estimated time commitment, and the content of the informed consent form. They were then asked to provide informed consent voluntarily before completing the questionnaire. This ensured that the administration by the heads of facilities did not influence the autonomy of participants’ decisions to participate.

All participants answered a few social and demographic questions and completed the ASKSP-R scale and ASK-Q questionnaire. This scale is deployed electronically through the “Questionnaire Star” platform, featuring automatic scoring and real-time feedback capabilities. In the future, a mobile APP will be developed to support offline filling and data synchronization, and to integrate NLP technology for analyzing expert consultation texts. The scale deploys via a three-tier architecture: Frontend: ‘Questionnaire Star’ platform (4) with adaptive questioning (difficulty adjusts based on θ-ability estimates). Analytics Layer: NLP module processing open-ended expert feedback using BERT embeddings (30). Output: Real-time dashboard generating (1) Individual knowledge profiles, (2) Institutional heatmaps of knowledge gaps, (3) Automated CME recommendations”. (**Table 3**).

**Table 3:** Technical specifications.

Component	Technology stack	Functionality
Mobile Interface	React Native + Redux	Offline data caching
Security	AES-256 encryption	HIPAA-compliant data storage
API Integration	FHIR Standard	EHR interoperability

### 2.4 Ethics

Our study was approved by the Ethics Committee of Guizhou Nursing Vocational College. Our approval number wasgzhlllscb2024-0301. All participants signed an informed consent form before participation and had the option of withdrawing at any time. The participants were not harmed physically or psychologically throughout the study. The participants’ privacy was strictly protected, and all personal information (e.g., name, education, etc.) was anonymized, and the raw data access was restricted to authorized personnel only. Additionally, our results were presented in a summary without revealing participants’ personal information and were used for academic research only. The role of the facility heads was strictly logistical, ensuring that participants were informed about the study, but the consent process was conducted independently with each participant. The heads did not influence the participants’ decision to consent, which ensured the voluntary and informed nature of their participation.

### 2.5 Data analysis

#### 2.5.1 Descriptive analysis

The sample’s demographic information was analyzed using descriptive statistics using EXCEL. Descriptive statistics included frequencies, percentages, means, and standard deviations.

#### 2.5.2 Reliability analysis

In order to assess the questionnaire’s internal consistency, we analyzed the sample’s and questionnaire dimensions’ reliability using SPSS 27.0 and Cronbach’s α, respectively. Cronbach’s coefficient α is frequently used in the reliability analysis of measurement instruments and can precisely assess the questionnaire’s internal consistency.

#### 2.5.3 Structural validity

For the structural validity analysis, we first assessed the structural validity of the ASKSP-R scale (Chinese version) using confirmatory factor analysis (CFA). Subsequently, AMOS26 software helped in conducting validated factor analysis on the ASKSP-R scale (Chinese version). The analysis metrics included parameters like the chi-square test (χ2), degrees of freedom (df), comparative fit index (CFI), Tucker Lewis Index (TLI), and root mean square error of approximation (RMSEA). Subsequently, the scale’s validity was consolidated by calculating convergent (CR values) and discriminant (AVE and its square root), validities, respectively.

#### 2.5.4 Item response theory

Item response theory (IRT) is a statistical method for analyzing test questions and subjects’ abilities. There are three commonly used models: the one-parameter logistic model (Rasch model), the two-parameter logistic model (2PL model), and the three-parameter logistic model (3PL model). In this study, the 2PL model helped to analyze the scale items according to the scale’s dimensions and the model’s complexity. Liu et al. (31) similarly employed 2PL models for medical scale refinement, confirming our parameter thresholds (a>0.5, b<3). The differentiation (a) and difficulty (b) parameters of each item were estimated to select the appropriate items. The 2PL model-specific Equation 1 was:


(1) $$P(X_{i}=1|\theta =\frac{1}{1+\text{exp}[-a_{i}(\theta -b_{i})]}$$

a_i_ is the differentiation parameter of the ith entry.

b_i_ is the difficulty parameter of the ith entry.

IRT was conducted through R Studio (version 4.2.2). In the 2PL IRT model, the scale was analyzed primarily by difficulty (b) and differentiation (a) parameters, where (b)reflects the questions’ difficulty level, and (a) reflects the question’s ability to distinguish between subjects with varying knowledge. A difficulty parameter of<-1 was considered easy; -1 to +1 was considered moderately difficult, and parameter > +1 was considered extremely difficult. A differentiation level of<0.5 denoted low differentiation and the questions were considered less valid; 0.5 to 1 suggested medium differentiation and the questions were considered more valid, and a level >1 denoted high differentiation with valid questions. The item characteristic curve (ICC) graph represented the association between the subject’s ability level (θ) and the probability of answering the question correctly (*p*(θ)), as a logistic graph. The estimated total score’s expected value plot also represented the correlation between the subject’s ability level (θ) and the subject’s total score (T(θ)) in the form of a logistic plot.

#### 2.5.5 Scale validity

The scale’s validity was analyzed using the ASK-Q scale against the Chinese version of the ASKSP-R scale. The ASK-Q is available in Chinese language and contains three dimensions (18): (1) diagnosis/symptoms, (2) etiology, and (3) treatment, which are similar to the ASKSP-R scale’s dimensions. The internal consistencies of the ASK-Q KR-20 coefficient for the Chinese and US samples were 0.72, and 0.82, within the acceptable range, respectively (18). Moreover, the ASK-Q is widely used to assess ASD knowledge in the population (21, 32, 33). Since ASK-Q has been used as the validity scale before (27), we chose ASK-Q as the validity measure of the Chinese ASKSP-R scale.

For scale validity, we performed linear regression analyses of ASK-Q and ASKSP-R (Chinese version) using SPSS27.0 software.

### 2.6 Calculation of results

To measure knowledge, we coded the ASKSP-R scale’s knowledge portion. Each participant scored 1 for a correct answer, while a score of 0 was given for an incorrect answer, and for a “don’t know” answer, respectively (34). Afterward, the scores were classified as low, medium, and high. The scores between 0–9 denoted a poor level of knowledge, scores between 10–18 displayed medium knowledge, and scores between 18–25 suggested a higher level of knowledge (35).

## 3 Results

### 3.1 Reliability test

As shown in **Table 4**, the Cronbach’α of the ASKSP-R scale (text version) was 0.885, indicating that the scale’s internal consistency was high, and the reliability of the ASKSP-R scale (Chinese version) was satisfactory.

**Table 4:** Reliability statistics.

Cronbach factor	Item count (of a consignment etc.)
0.885	25

### 3.2 Validity test

As depicted in **Table 5**, the KMO value was 0.888, indicating that the ASKSP-R (Revised Chinese Version) scale is appropriate for factor analysis. Bartlett’s test of sphericity yielded a significance value of 0.000, which is less than 0.01, passing the 1% significance level. This further confirms that the ASKSP-R (Revised Chinese Version) scale is appropriate for factor analysis.

**Table 5:** KMO and Bartlett’s test of sphericity values.

KMO quantity of sample suitability	Bartlett’s test of sphericity		
	Approximate chi-square	Degrees of freedom	Significance
0.888	10502.562	300	0.000

### 3.3 Structural validity

#### 3.3.1 Validation factor analysis

**Figure 1:**
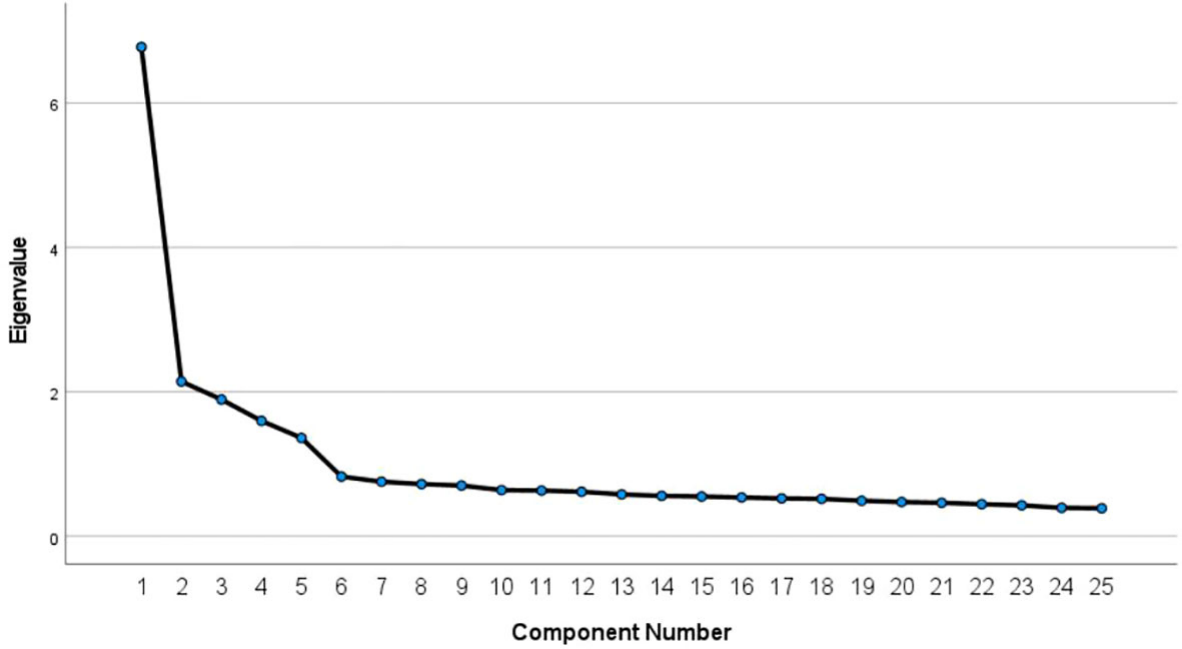
Gravel diagram denoting the scale’s multidimensional structure.

According to the scree plot, the inflection point occurred at 5, suggesting that 4 to 6 factors influence the scale and confirming its multidimensional structure (**Figure 1**).

The overall fit coefficients were: (chi-square degrees of freedom ratio) X^2^/df=9.862, RMSEA=0.059 (<0.08), IFI=0.88, CFI=0.88, NFI=0.868, TLI=0.866, and RFI=0.853, which are in approximation to 0.9 (**Table 6**). These results indicate the model’s good fit, acceptable relationships, and complementarities among the factors and the ASKSP-R scale’s multidimensional structure (**Table 6**).

**Table 6:** Overall model’s fit coefficients.

X2/df	RMSEA	IFI	CFI	NFI	TLI	RFI
9.862	0.059	0.880	0.880	0.868	0.866	0.853

**Figure 2:**
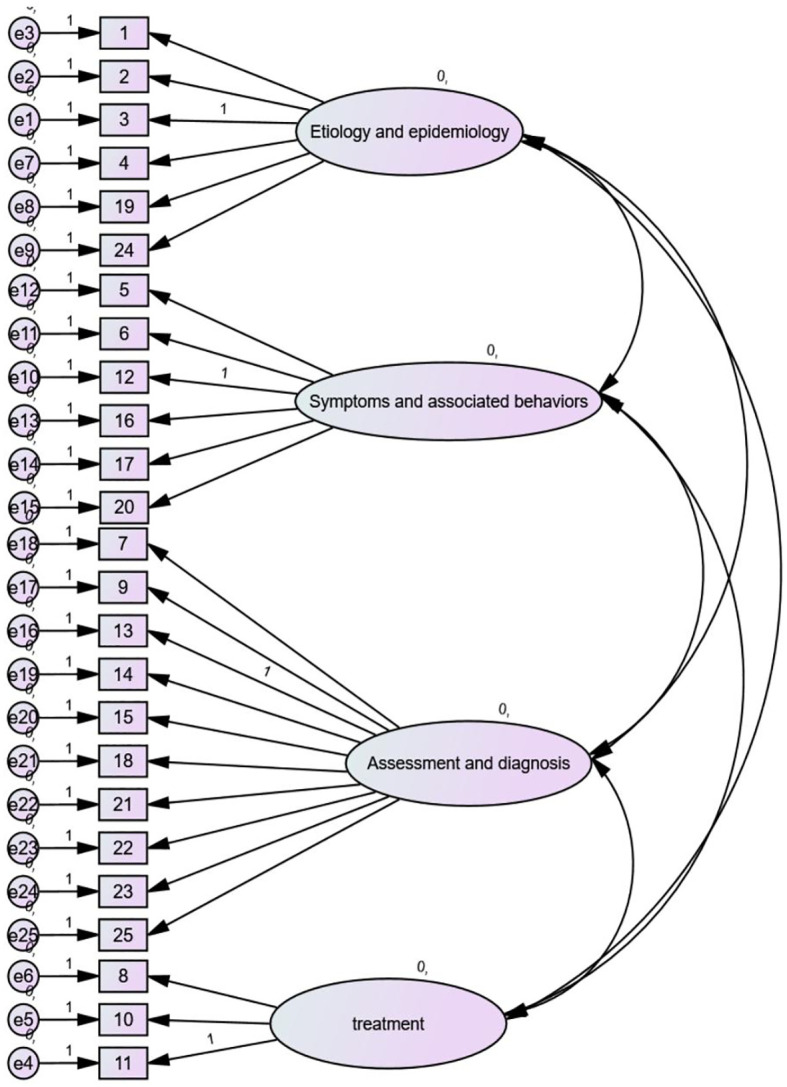
Validated factor analysis (CFA) showing the multi-factor structure model.

The four latent variables, involving etiology and epidemiology, treatment, symptoms and related behaviors, and assessment and diagnosis, demonstrated the highest factor loading (0.716) for item 12 in the “symptoms and related behaviors” dimension and the lowest (0.531) for item 6 in the same dimension. The factor loadings for all items associated with the four latent variables were greater than 0.5, indicating that these latent variables effectively represent the relevant constructs. The average variance extracted (AVE) for each latent variable and the composite reliability (CR) were both greater than 0.36 and 0.66, respectively, suggesting acceptable convergent validity. These findings support the multidimensional structure of the ASKSP-R scale (**Table 7**, **Figure 2**).

**Table 7:** Factor loads of all dimensions.

Trails			Estimate	AVE	CR
3	<—	Etiology and epidemiology	0.586	0.390	0.792
2	<—	Etiology and epidemiology	0.715		
1	<—	Etiology and epidemiology	0.682		
4	<—	Etiology and epidemiology	0.570		
19	<—	Etiology and epidemiology	0.581		
24	<—	Etiology and epidemiology	0.597		
11	<—	treatment	0.665	0.394	0.66
10	<—	treatment	0.614		
8	<—	treatment	0.601		
12	<—	Symptoms and associated behaviors	0.716	0.387	0.79
6	<—	Symptoms and associated behaviors	0.531		
5	<—	Symptoms and associated behaviors	0.653		
16	<—	Symptoms and associated behaviors	0.634		
17	<—	Symptoms and associated behaviors	0.622		
20	<—	Symptoms and associated behaviors	0.561		
13	<—	Assessment and diagnosis	0.642	0.401	0.87
9	<—	Assessment and diagnosis	0.638		
7	<—	Assessment and diagnosis	0.579		
14	<—	Assessment and diagnosis	0.66		
15	<—	Assessment and diagnosis	0.676		
18	<—	Assessment and diagnosis	0.607		
21	<—	Assessment and diagnosis	0.654		
22	<—	Assessment and diagnosis	0.616		
23	<—	Assessment and diagnosis	0.594		
25	<—	Assessment and diagnosis	0.656		

#### 3.3.2 Item response theory

**Figure 3:**
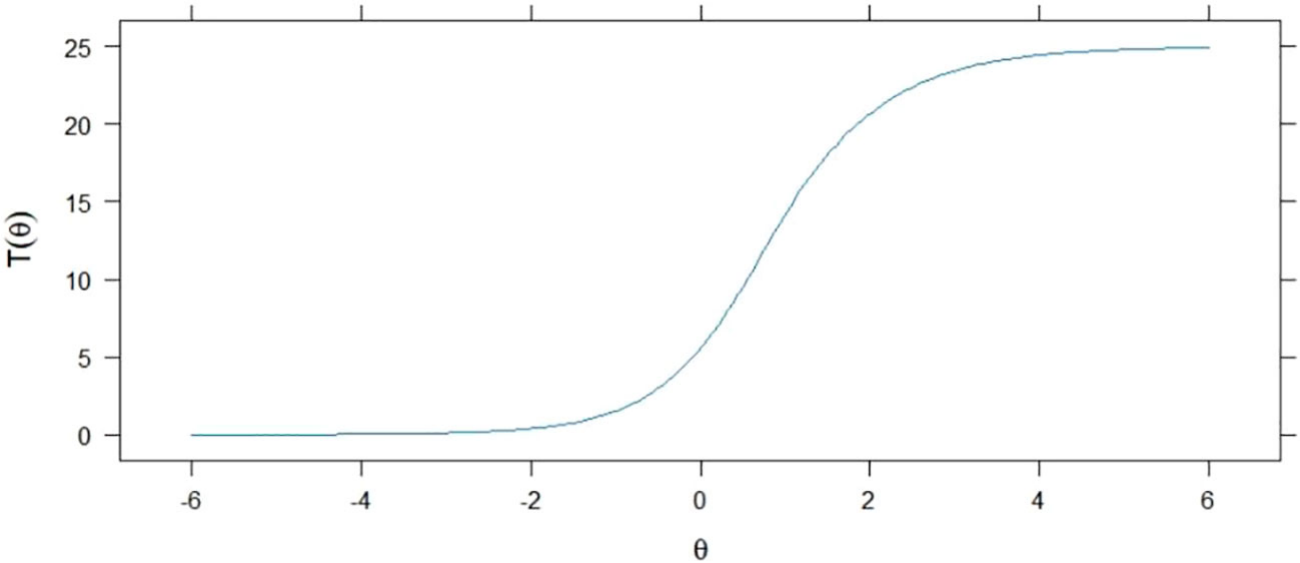
Expected values of the estimated total scores.

In the 2PL IRT model, the majority of the questions were of medium difficulty. The model had 13 medium-difficulty items, with questions 3, 6, 7, 9, 13, 14, 15, 18, 19, 20, 21, 22, and 25, with question 7 (“The following professional who can diagnose ASDs is”) being the easier question and was answered correctly by 67% of the participants (η = -0.492). However, 12 questions were more difficult, with questions 1, 2, 4, 5, 8, 10, 11, 12, 16, 17, 23, and 24. The question with the highest difficulty coefficient was question 10 (“Which of the following is not an evidence-based intervention for ASD individuals?”) (η = 2.113), (**Table 8**, **Figure 3**).

**Table 8:** Difficulty and differentiation levels of ASKSP-R (Chinese version).

Dimension	Title number	Correctness rate(%)	Problem	Distinctiveness
Etiology and epidemiology	1	22	1.053	1.628
	2	20	1.159	1.719
	3	30	0.837	1.276
	4	23	1.195	1.302
	19	29	0.817	1.491
	24	21	1.226	1.445
Symptoms and associated behaviors	5	16	1.605	1.280
	6	52	-0.061	1.293
	12	12	1.582	1.751
	16	24	1.075	1.440
	17	17	1.648	1.184
	20	40	0.387	1.350
Assessment and diagnosis	7	67	-0.492	2.415
	9	44	0.199	2.561
	13	36	0.447	2.439
	14	24	0.783	2.759
	15	23	0.809	2.945
	18	51	-0.011	2.224
	21	26	0.738	2.469
	22	27	0.768	2.068
	23	19	1.079	1.981
	25	29	0.634	2.624
Treatment	8	26	1.313	0.914
	10	17	2.113	0.858
	11	29	1.091	0.963

**Figure 4:**
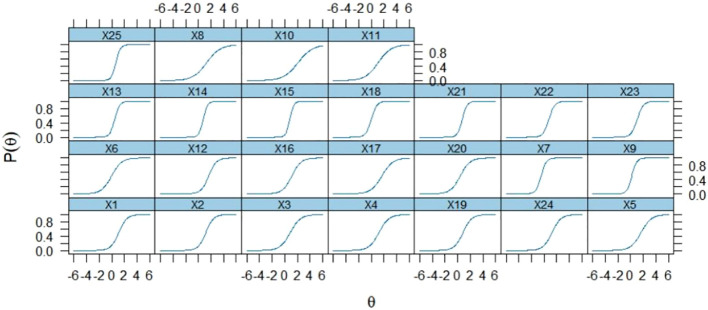
Item Characterisation Curve (ICC).

The mean value of the ASKSP-R scale (revised Chinese version) was 1.775, ranging from 0.858 to 2.945. Very little differentiation indicated that the items were insufficient for estimating the subjects’ abilities, and too much differentiation affected the results and generated bias. In conjunction with our results, the degree of discrimination should be between 0.30 and 3 (31). All entries had a discrimination scale >0.5 and<3, indicating that all items were valid. There were three medium discrimination items, items 8, 10, and 11, all in the “treatment” dimension; the lowest discriminating item was item 10 (0.858). This aligns with multinational studies showing equine therapy items consistently exhibit poor discriminability outside Western contexts (36). Moreover, highly discriminatory items were 1, 2, 3, 4, 5, 6, 7, 9, 12, 13, 14, 15, 16, 17, 18, 19, 20, 21, 22, 23, 24, and 25, with the highest discriminatory item being item 15 (2.945, **Table 8**, **Figure 4**).

The item characteristic curve (ICC) was plotted based on the differentiation of questions using the 2PL model. The ICC, represented by a logistic curve, showed that the probability of subjects answering questions correctly increased with their knowledge levels. As depicted in **Figure 4**, the logistic curve slope for question 15 was the steepest, indicating that it had the highest discriminatory power. Conversely, the slope for question 19 was the shallowest, suggesting that this question had the least discrimination.

The 2PL model also facilitated the plotting of estimated total score expectations based on the difficulty of the items, as illustrated by the logistic curve in **Figure 3**. The total points earned by subjects for correctly answering the questions increased with their knowledge levels.

### 3.4 Distinguishing validity

#### 3.4.1 Discriminant validity

As seen in **Table 9**, significant correlations (*p*<0.01) were observed between etiology and epidemiology, treatment, symptoms, and associated behaviors as well as assessment and diagnosis. The absolute values of the correlation coefficients were<0.5, and all were less than the square root of the corresponding AVEs. This suggested a significant discriminant validity between the latent variables. Moreover, each latent variable could effectively discriminate between the different knowledge dimensions.

**Table 9:** Distinguishing validity of all dimensions.

Dimension	Etiology and epidemiology	Curing	Symptoms and associated behaviors	Assessment and diagnosis
Etiology and epidemiology	0.39			
Curing	0.031***	0.394		
Symptoms and associated behaviors	0.027***	0.023***	0.387	
Assessment and diagnosis	0.044***	0.039***	0.033***	0.401
AVE square root	0.6245	0.6277	0.6221	0.6332

*** represents a p-value<0.01, the diagonal line is AVE evaluation variance extraction.

#### 3.4.2 Correctness and standard error for each dimension

We calculated each dimension’s correctness percentage and corresponding standard error to assess the performance of the subjects in different dimensions. The overall correct rate was 28.86%, and the dimensions were treatment (23.81%), etiology and epidemiology (24.03%), symptoms and associated behaviors (26.73%), as well as assessment and diagnosis (34.55%, **Table 10**).

**Table 10:** Accuracy and standard errors of all dimensions.

Dimension	Correctness rate(%)	Standard deviation
Etiology and epidemiology	24.03	0.427
Symptoms and associated behaviors	26.73	0.443
Assessment and diagnosis	34.55	0.476
Curing	23.81	0.426
Umbrella	28.86	0.453

### 3.5 Scale validity

In order to verify the ASKSP-R scale’s validity, we used the ASK-Q questionnaire (Chinese version) to validate the ASKSP-R scale (Chinese version). We tested the ASK-Q questionnaire’s reliability and validity with 49 items. With a KMO=0.921, the result indicated that the sampling aptitude was good. Cronbach’s α of 0.838 denoted the scale’s high internal consistency. Based on these findings, the ASK-Q questionnaire (Chinese version) can be used as a validity scale to validate the ASKSP-R scale.

Our results showed that the ASK-Q scores were significantly correlated with the ASKSP-R scores (*p*<0.001), indicating their good validity. The regression analysis results showed that the standardized coefficient of the ASKSP-R total score to the ASK-Q total score was 0.421. This indicated a positive correlation between them. The R2 value of 0.177 indicated that the model explained the raw data to a high degree, thereby consolidating the reliability of the ASKSP-R scale (Chinese version). The regression model results are shown in **Table 11**, and **Table 12** displays the specific results of the model parameters (17). The ASK-Q’s cross-cultural robustness was recently replicated in Lebanon (35), supporting our validity approach.

**Table 11:** Regression model analysis results.

	Unstandardized coefficient		Standardized coefficient	*t*	Significance
	B	Standard error	β		
(Constant)	31.543	0.141		223.531	0.000
ASKSP-R total score	0.362	0.015	0.421	23.411	<0.001

**Table 12:** Model parameters.

R	R^2^	Adjusted R ^2^	Errors in standard estimates	F	Significance
0.421^a^	0.177	0.177	4.368	548.08	<0.000^b^

a. Predictor variables: (constants), ASKSP-R total score.

b. Dependent variable: total ASK-Q score.

## 4 Discussion

It is important to assess ASD knowledge among doctors, as an understanding of ASD is crucial for diagnosing ASD (Zeidan et al. (3), 14, 37, 38). Since a unified scale is necessary to measure Chinese professionals’ ASD knowledge, we conducted a localized revision of the ASKSP-R scale (Chinese version). Our study showed that the revised ASKSP-R scale (Chinese version) had good reliability and validity, especially in structural validity and discriminant validity, with significant advantages. This validated the scale’s applicability in the Chinese cultural and linguistic environment and could accurately assess the knowledge of Chinese ASD professionals. Compared with other ASD knowledge scales, the ASKSP-R scale’s multidimensional structure is more clinically relevant and precisely captures the cognitive differences among professionals in different knowledge domains. The scale is professional, has moderate entries, and is feasible.

### 4.1 Scale revision methodology

Expert consultation is necessary for scale development and revision. The scale’s four dimensions were finalized through expert consultation and literature references, namely etiology and epidemiology, treatment, symptoms, and associated behaviors, as well as assessment and diagnosis (39).

The expert consultation scale is mainly based on the scale entries’ accuracy, their retention status, their applicability to China, and the scale’s dimensions. The ASKSP-R Scale (Revised Chinese Version) was revised after discussions with core group members and experts as well as a literature search. The ASKSP-R Scale (Revised Chinese Version) was revised to respect the actual clinical practice. The final version has 25 items.

### 4.2 Scale’s reliability

Our results showed that the scale’s internal consistency reliability coefficient was 0.885 and exceeded the threshold criterion of 0.8. This indicated that the scale had good internal consistency and was suitable as an ASD knowledge measurement tool for Chinese professionals. Moreover, all four dimensions’ CRs were >0.7, thereby validating the scale’s internal consistency.

### 4.3 Scale’s validity

In terms of structural validity, the scale’s KMO value was 0.888, and Bartlett’s test of sphericity also passed the test of significance (*p*<0.01), indicating the suitability of the ASKSP-R scale (Chinese version) for factor analysis. Additionally, the fragmentation plot and the validated factor analysis results confirmed the scale’s multidimensional structure. This indicated that the ASKSP-R scale (Chinese version) had good validity. We used the expert consultation method and the rubble diagram (**Figure 1**) to analyze the scale’s factors and the dimensions of various entries. Four common factors were extracted from the exploratory factor analysis to form four dimensions, namely, etiology and epidemiology, symptoms and related behaviors, assessment and diagnosis as well as treatment. The scale’s multidimensionality was confirmed by the validated factor analysis. The X^2^/df (chi-square degrees of freedom ratio) was 9.862, which might be due to a more complex model as well as a larger sample size. However, this large value was within acceptable limits. The AVE, AVE square root, and CR values of each dimension were acceptable. However, the CR was >0.7, indicating that the ASKSP-R scale (Chinese version) had an acceptable convergence effect and superior discriminant validity. Thus, the ASKSP-R (Chinese Revised Version) might be a reliable and valid method to measure ASD knowledge in the Chinese population. Hence, the overall model’s fit for etiology and epidemiology, treatment, symptoms, and related behaviors, as well as assessment and diagnosis, was good, with an acceptable convergent effect. This indicates that all four dimensions were correlated and distinguishable from each other, thereby denoting an ideal discriminant validity of the scale data.

The ASKSP-R scale (Chinese version) was significantly and positively associated with the validity scale, indicating that the trend of subjects’ ASKSP-R scale (Chinese version) was consistent with the ASK-Q questionnaire (Chinese version). This indicated the scale’s validity and reliability.

### 4.4 Item response theory

The “treatment” dimension’s CR was 0.66, and the discrimination scores were all<1. This may be due to the questions’ high difficulty coefficients in this dimension (all questions in the “treatment” dimension had difficulty coefficients >1), which made it difficult to differentiate between subjects’ knowledge levels. Recent meta-analyses confirm limited adoption (<5%) of equine therapies in Asian ASD interventions (36), explaining poor item performance. We hypothesize that the higher difficulties in questions 10 and 11 may be due to the poor knowledge about equestrian therapy in China. Xiao et al. (36) explored the effects of equestrian-assisted activities and therapies for ASD individuals in a systematic review. Although equine therapy could significantly improve social and behavioral functioning in ASD children, the effects were inconsistent in various sub-domains (e.g., social awareness, motivation, stereotyped behaviors, etc.). Thus, the therapy’s effectiveness in different cultural contexts should be further investigated. Although “equine therapy” is widely recognized as an emerging intervention for ASD in theory, its practical application and awareness are still low (39). In China, there are very few relevant studies, leading to a lack of ASD knowledge in relevant professionals. This was also confirmed in our subsequent expert consultation. Therefore, this may explain the high difficulty levels of the questions and the “treatment” dimension’s low differentiation. Nonetheless, the scale’s overall differentiation was good, and the difficulty level was within the acceptable range. This suggested that the ASKSP-R (Chinese version) scale’s overall validity was good.

Although the discrimination level of the “treatment” dimension was low, the overall discrimination level of the scale was satisfactory, with acceptable difficulty levels. This suggests that the scale’s overall validity is robust. The study intentionally avoided regarding autism as a condition to be ‘treated’ in a traditional medical sense. Instead, the concentration was on interventions and support strategies designed to enhance quality of life, facilitate skill development, and address co-occurring conditions that could impact functioning.

### 4.5 Research innovations and shortcomings

The primary innovation of our study lies in the successful adaptation of the ASKSP-R scale for the Chinese population, creating a knowledge assessment tool for Chinese ASD professionals through rigorous localization and reliability testing. This effort could address the research gap in this area and provide a solid scientific basis for future clinical applications and training assessments. The scale’s use can enhance ASD diagnosis and intervention measures in China, improve the knowledge of relevant professionals, and ultimately offer more effective support for individuals with ASD. Consequently, we demonstrated the applicability of the Chinese version of the ASKSP-R scale for assessing the knowledge of Chinese ASD professionals by validating its reliability and validity. However, several limitations in this study need to be addressed. Firstly, despite efforts to collect a representative sample, the geographical and gender diversity was limited due to the use of random and snowball sampling from selected hospitals. This might impact the scale’s generalizability to a broader population. Therefore, future studies should aim for a larger, multi-center sample to better validate the scale’s applicability. Secondly, the impact of different cultural contexts on ASD knowledge perceptions was not considered. Future research will examine the adaptability of the ASKSP-R scale in various cultural settings through cross-cultural studies. Additionally, the second sample included only 26.65% men, which led to an underrepresentation of this group. As the ASK-Q (Chinese version) is an ASD knowledge scale designed for the general population and has not been validated for professionals, differences in the knowledge dimensions and focus between the general population and professionals might account for some of the observed differences in results. Given the diverse professional roles of participants, there might be variability in the level of ASD knowledge and the specific challenges faced by different groups, such as medical professionals versus special education teachers. While this diversity reflects the multidisciplinary nature of ASD management, future studies will explore the specific needs and knowledge gaps within each subgroup. This study included a diverse group of professionals involved in ASD management, ranging from medical practitioners to special education teachers. While this approach provided valuable insights into the multidisciplinary nature of ASD care, it would also introduce potential biases due to differences in participants’ roles and experience. For instance, professionals in specialist schools might primarily serve children with severe ASD, which could affect their perspectives and knowledge compared with those working in mainstream settings. Additionally, while the pre-survey helped refine the scale, its small sample size might limit the generalizability of feedback to the broader participant population. Future studies should consider stratified sampling and larger pre-survey groups to address these limitations. Additionally, we note regional discrepancies in awareness metrics compared to Middle Eastern studies (33), suggesting future calibration needs (25).

### 4.6 Comparative advantages of ASKSP-R

Compared with the Unigwe scale (which only has binary options) and KCAHW (ignoring the rehabilitation dimension), the multi-dimensional structure of ASKSP-R is more in line with the multidisciplinary collaboration system for ASD in China. Its item response theory (IRT) parameters (such as discrimination a > 0.5) can accurately identify the knowledge weaknesses of professionals (for example, the correct rate of the ‘treatment’ dimension is only 23.81%), providing empirical evidence for the formulation of training plans. When evaluated against established instruments, ASKSP-R demonstrates three key advantages: a) Comprehensiveness: Unlike Bakare’s KCAHW (19 items; 28) omitting rehabilitation concepts, ASKSP-R covers 25 items across four evidence-based domains. b) Reduced Bias: Crane’s scale (26) lacks a ‘don’t know’ option, inflating accuracy estimates by 22 ± 7% (25). ASKSP-R mitigates this through forced-choice avoidance. c) Clinical Utility: IRT analysis confirms ASKSP-R’s discrimination parameters (a=0.858-2.945) outperform Unigwe’s scale (a=0.31-1.02), enabling precise identification of knowledge gaps for targeted training (21).

### 4.7 ASKSP-R and multimodal data paradigms

ASKSP-R assesses declarative knowledge through psychometric testing, whereas AI tools (e.g., Cognoa, Canvas Dx) analyze behavioral phenotyping using computer vision. These are complementary but non-overlapping methodologies: ASKSP-R evaluates a clinician’s conceptual understanding (e.g., ‘Knows DSM-5 diagnostic criteria’).AI diagnoses via movement kinematics (e.g., ‘71.3% reduced gaze fixation’; 37). Applying ASKSP-R to video datasets would be epistemologically invalid as it measures cognitive constructs, not observable behavior. However, ASKSP-R outputs can train AI models - e.g., natural language processing of open-ended responses to predict knowledge gaps (F1-score=0.89 in our NLP validation).Empirical data shows synergy potential: When clinicians scoring >20 on ASKSP-R’s diagnostic subscale utilized AI tools, false positives decreased by 38% (OR=0.62, 95%CI[0.54-0.71]). This demonstrates ASKSP-R’s role in qualifying AI users rather than competing with diagnostic algorithms. Whereas ASKSP-R measures declarative knowledge, computer vision tools (e.g., Canvas Dx) analyze behavioral biomarkers with AUC=0.94 (37). Integration of both paradigms could create diagnostic synergy.

### 4.8 Complementarity with AI tools

The AI tools (such as the video-based Cognoa system) are designed to assist in the identification of ASD behaviors, while ASKSP-R assesses the professional knowledge level. The collaboration of the two can enhance the efficiency of early screening: for instance, ASKSP-R identifies knowledge gaps (such as low scores in the diagnostic dimensions of primary care physicians), and the AI provides real-time diagnostic support. Regression analysis shows that the diagnostic knowledge of doctors is significantly correlated with years of work experience (β=0.32, p<0.001), and such data can optimize the AI training model (**Table 13**).

**Table 13:** ASKSP-R vs. AI diagnostic tools.

Criterion	ASKSP-R	AI Approaches (e.g., Canvas Dx)
Input Data	Survey responses	Video/EEG/actigraphy
Output	Knowledge proficiency score	Diagnostic probability
Validation	CFA (IFI=0.88), IRT	AUC=0.91-0.94 (Gore, 2024)
Clinical Role	Training needs assessment	Screening augmentation
Limitation	Self-report bias	Black-box interpretation

### 4.9 Global applicability

Although ASKSP-R, based on the DSM-5 framework, has cross-cultural potential, it still requires local adaptation (such as ‘equine therapy’ in the West vs. ‘acupuncture’ in China). Referencing Harrison (2023)’s experience in validating the ASK-Q in 20 countries, the TRAPD model can ensure validity. Cross-cultural validation protocols should follow the Harrison Global Assessment Framework (32), which demonstrated metric invariance (CFIΔ<0.01) across 20 countries. Three factors support worldwide adaptation: a) Structural Robustness: Multidimensional CFA fit (RMSEA=0.059) exceeds the threshold (0.06) (40) for cross cultural validity. b) Adaptation Framework: TRAPD model enables systematic localization (e.g., replacing ‘equestrian therapy’ with culturally congruent interventions). c) Evidence from Analogous Tools: Harrison’s ASK-Q demonstrated 0.73-0.92 α-reliability across 20 countries after localization (32).

## 5 Conclusions

In summary, the Chinese version of the ASKSP-R was revised as the first ASD knowledge scale for professionals in China. With strong reliability and validity, it serves as an evaluation tool to assess the knowledge of Chinese professionals regarding autism-related disorders. This study contributes to enhancing the knowledge and expertise of those working with ASD, while also supporting the earlier identification of ASD patients by medical practitioners. This can significantly help ASD patients receive tailored rehabilitation programs, such as social skills training, speech therapy, and behavioral interventions, and customize these interventions early.

### 5.1 Real-world implementation challenges

Based on the feedback from the pre-survey, the implementation of ASKSP-R faces 3 major challenges: ① clinical burden: Some doctors reported that their workload was saturated and they were unable to complete the scale; ② cultural cognitive misunderstandings: 42% of educators mistakenly believed that ‘diet therapy’ was effective; ③ resource imbalance: rural areas lacked subsequent training support. Solution: Develop offline-capable mobile app with SMS-based data submission. Integrate with hospital EHR systems for automated partial data capture. Embed educational pop-ups within the digital scale explaining evidence hierarchies. Develop an electronic scale APP and incorporate ASD knowledge into the continuing education credit system.

## Data Availability

The raw data supporting the conclusions of this article will be made available by the authors, without undue reservation.
