# Supplementary material for: Localization of the Autism Spectrum Disorder Knowledge Scale Professional Version (ASKSP-R) in western cities of China
Source: Front Psychiatry. 2025 Sep 10;16:1550823. doi: 10.3389/fpsyt.2025.1550823 (PMC12457664; doi:10.3389/fpsyt.2025.1550823)
Supplement: Supplementary file 1 [file Supplementaryfile1.docx]

**Modifications of ASKSP-R Chinese Version Scale**

Question 1: Since there are no statistics on the population affected by autism spectrum disorders in China, referred to the China Institute for Public Welfare, "the percentage of the affected population of ASD is ?" is replaced by "The current morbidity of autism in China is approximately ?." Revise the answer accordingly.

Question 4: Since the concept of race is not formally defined and recognised in Chinese society, refer to the expert opinion for localisation and change the answer to "a. Consistent among different ethnic groups; b. Inconsistent among different ethnic groups, Han children are diagnosed more frequently and earlier than other children of different ethnic groups; c. Inconsistent among different ethnic groups, children from different ethnic backgrounds are diagnosed more frequently and earlier than children of Han; d. Prevalence in different ethnic groups is unknown; e. Don't know".

Question 7: Since there are no "occupational psychologists" or "occupational therapists" in China, and "psychologists" are the main group of people who diagnose ASD in the Chinese context. According to the experts' opinions, taking into account China's national conditions, we amend "a. Occupational psychologists" to "a. Psychologists" and "d. Occupational therapists" to "d. Psychologists". "d. Psychological counsellors".

Question 8: Since China has not yet specified what kind of ASD children should be admitted to special schools, according to the experts' opinions and with reference to the "Specification for Autism Screening and Intervention Services for Children 0-6 Years of Age (for Trial Implementation)" issued by the National Health and Health Commission, the question will be revised to: "8. Which of the following is an incorrect description of the places of intervention for children with autism spectrum disorders. " The option is amended as "a. For children within 1.5 years of age, family intervention can be provided under the guidance of professional medical institutions; b. For children between 1.5 and 3 years of age, professional medical institutions can be chosen to provide rehabilitation training and family intervention at the same time; c. For children after 3 years of age, those who are relatively mildly ill and have certain social interaction and communication skills can receive integrated education in ordinary kindergartens while combining with training in professional institutions; d. After the age of 3, children with relatively severe conditions and weak social interaction and communication skills can receive integrated education in professional medical institutions, special education institutions or ordinary kindergartens; e. Don't know".

Question 11: According to expert opinion and literature , change "sensory integration therapy" to "restricted (e.g. running) exercise".

Question 13: According to expert advice and Chinese localization, the "English" in the answer is changed to "Chinese".

Question 21: Based on expert opinion and localisation, replace "US" with "China" and change the options to "a. Childhood Autism Rating Scale (CARS); b. Integrated Visual and Auditory Continuous Performance Test (IVA); c. Autism Behavior Checklist (ABC); d. Peabody Picture Vocabulary Test (PPVT); e. Don't know".

All of the above changes have been made with the consent of McClain, the author of ASKSP-R.
